# Thiazolidinedione Derivatives: In Silico, In Vitro, In Vivo, Antioxidant and Anti-Diabetic Evaluation

**DOI:** 10.3390/molecules27030830

**Published:** 2022-01-27

**Authors:** Manal Y. Sameeh, Manal M. Khowdiary, Hisham S. Nassar, Mahmoud M. Abdelall, Hamada H. Amer, Abdelaaty Hamed, Ahmed A. Elhenawy

**Affiliations:** 1 Chemistry Department, Faculty of Applied Science, Umm El Qura Branch, Makkah 24211, Saudi Arabia; Myassemih@uqu.edu.sa (M.Y.S.); mmkhowdiary@uqu.edu.sa (M.M.K.); 2 Applied Surfactant Laboratory, Egyptian Petroleum Research Institute, Nasr City, Cairo 11727, Egypt; 3 Department of Chemistry, Faculty of Science and Arts in Al-Mukhwah, Al-Baha University, Al Bahah 65311, Saudi Arabia; h_nassar5@yahoo.com; 4 Chemistry Department, Faculty of Science, Al-Azhar University, Nasr City, Cairo 11884, Egypt; abdelall_sci@yahoo.com (M.M.A.); abdelaatyhamed.1@azhar.edu.eg (A.H.); 5 Department of Animal Medicine and Infectious Diseases, Faculty of Veterinary Medicine, University of Sadat City, Sadat City 32958, Egypt; dr.hamada1435@gmail.com or; 6 Department of Chemistry, Turabah University College, Taif University, P.O. Box 11099, Taif 21944, Saudi Arabia

**Keywords:** thiazolidinediones, anti-diabetic, antioxidant, molecular docking

## Abstract

This work aimed to synthesize a new antihyperglycemic thiazolidinedione based on the spectral data. The DFT\B3LYP\6-311G** level of theory was used to investigate the frontier molecular orbitals (FMOs), chemical reactivity and map the molecular electrostatic potentials (MEPs) to explain how the synthesized compounds interacted with the receptor. The molecular docking simulations into the active sites of PPAR-*γ* and *α*-amylase were performed. The in vitro potency of these compounds via *α*-amylase and radical scavenging were evaluated. The data revealed that compounds (**4**–**6**) have higher potency than the reference drugs. The anti-diabetic and anti-hyperlipidemic activities for thiazolidine-2,4-dione have been investigated in vivo using the alloxan-induced diabetic rat model along with the 30 days of treatment protocol. The investigated compounds didn’t show obvious reduction of blood glucose during pre-treatments compared to diabetic control, while after 30 days of treatments, the blood glucose level was lower than that of the diabetic control. Compounds (**4**–**7**) were able to regulate hyperlipidemia levels (cholesterol, triglyceride, high-density lipoproteins and low- and very-low-density lipoproteins) to nearly normal value at the 30th day.

## 1. Introduction

**Figure 1:**
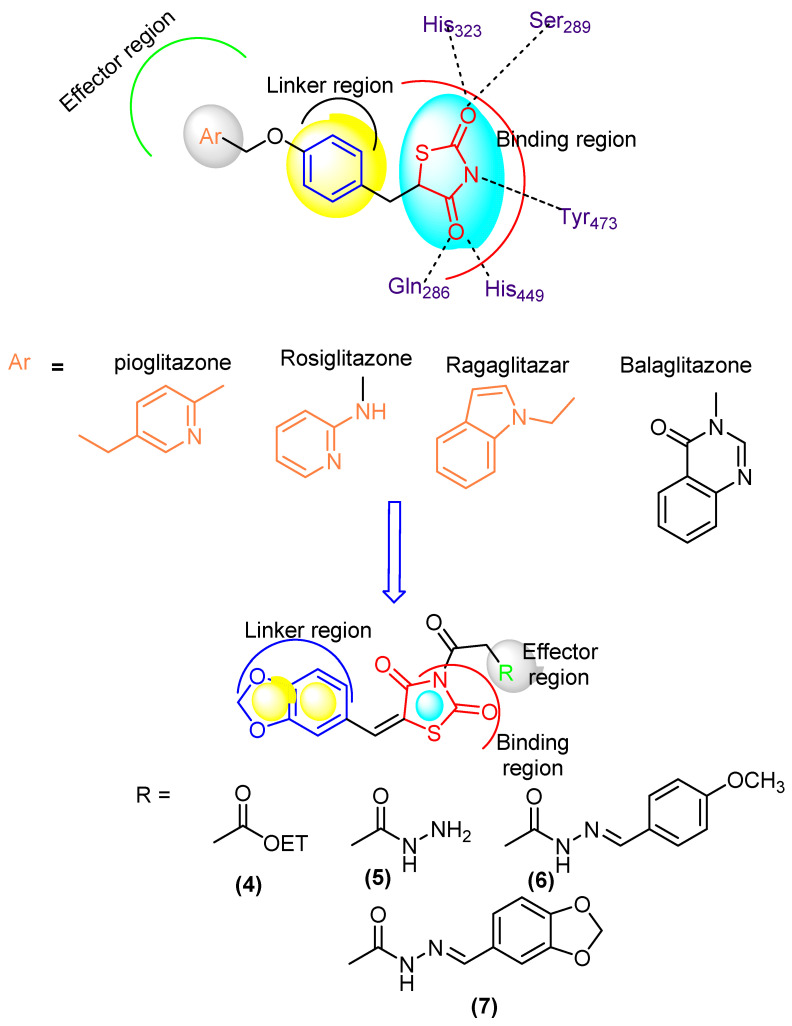
Designed molecule compliance of Rosiglitazone pharmacophore.

Diabetes mellitus (DM) is a common chronic health problem and around 416 million people worldwide are suffering from it. This figure is expected to reach 618 million cases by 2040 [1,2,3,4]. DM was classified into two major classes: “DMI” and “DMII” [5,6]. More than 90% of cases are diagnosed with DMII (non-insulin dependent diabetes mellitus; NIDDM). DMII is recognized by tissue resistance to the action of insulin combined with a reduction in insulin secretion because of resistance or deficiency against a beta cell, that requires insulin to control the disease [6]. Increasing glucose levels are related to raising several health problems such as cardiovascular disease, extra damage via insulin, etc. The enzyme *α*-glucosidase is vital in the therapeutic process and is found in secretions from salivary glands and the pancreas [7]. It belongs to the endoamylases family and is responsible for the breakdown of the *α*-*D*-(1,4) glycosidic bond in starch to obtain monosaccharides [8]. Inhibition of this enzyme is an effective protocol to reduce blood glucose level [9]. DMII patients have a two to fourfold higher risk of cardiovascular disease than non-DM patients [9]. Thiazolidinediones (TZDs) are an efficient DMII drug type [10] (Figure 1). Several TZDs’ derivatives have been marketed for the treatment of DII such as pioglitazone, rosiglitazone, ragaglitazar, balaglitazone [11] (Figure 1). Clinical studies showed that mixed therapy between *α*-glucosidase inhibitors and PPAR-*γ*-agonists (peroxisome proliferator-activated receptor [11,12,13]) was a helpful strategy for treatment of DMII, as acarbose combined with pioglitazone, the first prevents NIDDM and reduces the cardiovascular problem [14]) and the second acting on cardiovascular action and anti-atherosclerosis [15]. 

The combination thereby is also effective for older patients with hypertension and DMII [16], as well as induced-electrolyte-disturbance in DMII rats [17]. Despite the efficiency of the present drugs such as acarbose and TZDs, they can cause adverse effects such as diarrhea, hepatotoxicity, carcinogenesis, oedema, cardiac side effects and obesity [18,19]. The search for more effective novel drugs with reduced side effects is highly demanded [20]. Recently, unimolecular with multi-target drugs have been developed to handle metabolic-syndrome, as an inhibitor of Na-dependent-glucose-transporters “SGLTI” [21,22].

The 2,4-Thiazolidinediones have several potential uses as anti-cancer [23], antioxidant [24], anti-malarial [25], anti-obesity and anti-microbial agents [25,26]. In view of the above mentioned, we have reported new thiazolidinediones and evaluated their properties in silico using molecular docking and in vitro testing as α-glucosidase inhibitors and antioxidant agents, as well as studying the anti-diabetes efficacy in vivo.

## 2. Materials and Methods

The melting points, thin layer chromatography, IR, NMR, elemental analyses (C, H, N), and mass spectra were recorded (Supplementary Materials).

### 2.1. Chemistry 

#### 2.1.1. Ethyl-2-(5-(benzo[d][1,3]dioxol-5-ylmethylene)-2,4-dioxothiazolidin-3-yl)acetate (**4**)

Potassium salt **3** (0.1 mol) was refluxed with ethyl bromoacetate (0.1 mol) in DMF to give ester **4**. The crude product was separated and crystallized from EtOH to give ester **4** as pale-yellow crystals, yield 74%, m.p. 170–172 °C. IR (KBr): (ν/cm^−1^) = 1692, 1736 (C=O). ^1^H-NMR (500 MHz, DMSO-*d_6_*) spectra δ_H_ (ppm) = 7.79 (s, 1H, CH=benzylidine), 7.20–7.14 (m, 1H, Ar-H), 7.10 (dd, *J* = 1.9, 0.5 Hz, 1H, Ar-H), 7.02 (d, *J* = 8.9 Hz, 1H, Ar-H), 6.01 (s, 2H, 2H, CH_2_ of dioxole ring), 4.73 (s, 2H, NCH_2_), 4.18 (q, *J* = 6.6 Hz, 2H, CH_3_CH_2_), 1.24 (t, *J* = 6.6 Hz, 3H CH_3_). ^13^C NMR (DMSO-*d_6_*) spectra δ_C_ (ppm) = 170.18, 168.29, 167.98, 149.34, 148.33, 130.72, 128.12, 125.32, 119.76, 110.52, 109.65, 101.85, 61.46, 42.42, 14.17. Anal. Calc’d for C_15_H_13_NO_6_S (335): C, 53.73; H, 3.88; N, 4.17. Found: C, 53.71; H, 3.84; N, 4.13.

#### 2.1.2. 2-(5-(Benzo[d][1,3]dioxol-5-ylmethylene)-2,4-dioxothiazolidin-3-yl)acetohydrazide (**5**)

A mixture of ester **4** (0.1 mol) stirred with hydrazine hydrate (0.1 mol) in EtOH (30 mL) for 3 h. at 160 °C, then cooled, filtered, and crystallized from acetic acid to give ester **5** as white crystals, yield 65 %, m.p. 240–242 °C. IR (KBr): (ν/cm^−1^) = 3385, 3251, 3147 (NH_2_, NH), 1681, 1743 (C=O). MS *m*/*z* (%): 425 (M ^+^, 19.3%). ^1^H NMR (500 MHz, DMSO-*d_6_*) δ_H_ (ppm) = 8.73 (t, *J* = 4.1 Hz, 1H, NH), 7.81–7.77 (m, 1H, CH= benzylidene and Ar-H), 7.20–7.14 (m, 1H, Ar-H), 7.10 (dd, *J* = 1.9, 0.5 Hz, 1H, NH_2_), 7.02 (d, *J* = 8.9 Hz, 1H, Ar-H), 6.01 (s, 2H, CH_2_ of dioxole ring), 4.55 (s, 2H, NCH_2_). ^13^C NMR (125 MHz, DMSO) δ_C_ (ppm) = 169.90, 168.76, 168.43, 149.34, 148.33, 130.68, 128.12, 125.32, 119.76, 110.52, 109.65, 101.85, 42.66. Anal. Calc’d for C_13_H_11_N_3_O_5_S (321): C, 48.59; H, 3.42; N, 13.08. Found: C, 48.55; H, 3.38; N, 13.02.

#### 2.1.3. General Procedure for Synthesis of Hydrazones **6** and **7**

A mixture of hydrazide **5** (0.1 mol) and aromatic aldehydes (0.1 mol) in EtOH (30 mL) was heated for 3 h. The mixture was cooled, then filtered the solid product, and crystallized by the proper solvent.

#### 2.1.4. 5-(Benzo[d][1,3]dioxol-5-ylmethylene)-3-(2-(2,4-dioxo thiazolidin-3-yl) acetyl) thiazolidine-2,4-dione (**6**)

Hydrazide **5** (0.01 mol) and piperonal (0.01 mol) in ethanol (50 mL) were refluxed for 3 h. and crystallized from EtOH/benzene to give hydrazide **6** (61%) as yellow crystals, m.p. 202–204 °C. IR (KBr): (ν/cm^−1^) = 3142 (NH),1689, 1736 (C=O). ^1^H NMR (DMSO-*d_6_*) spectra δ_H_ (ppm) = 9.65 (s, 1H, NH), 7.81–7.77 (m, 1H, CH = benzylidine), 7.69 (t, *J* = 0.5 Hz, 1H, CH=N), 7.30–7.02 (m, *J* = 8.9 Hz, 5H, Ar-H), 6.93 (d, *J* = 8.3 Hz, 1H), 6.01 (d, *J* = 5.0 Hz, 2H, dioxole ring), 4.85 (s, 2H, dioxole ring). ^13^C NMR(DMSO-*d_6_*) spectra δ_C_ (ppm) = 169.93, 168.41, 165.85, 149.34, 149.01, 148.29 (d, *J* = 9.2 Hz), 145.49, 130.68, 128.12, 127.95, 125.32, 121.59, 119.76, 110.52, 109.65, 108.41, 105.98, 101.85, 101.38, 42.01. Anal. Calc’d for C_21_H_15_N_3_O_7_S (453): C, 55.62; H, 3.31; N, 9.27. Found: C, 55.58; H, 3.26; N, 9.23.

#### 2.1.5. 2-5-(Benzo[d][1,3]dioxol-5-ylmethylene)-2,4-dioxothiazolidin-3-yl)-N′-4-methoxy-benzylidene) acetohydrazide (**7**)

Hydrazide **5** (0.01 mol) and *p*-anisaldehyde (0.01 mol) in ethanol (50 mL) and refluxed for 3 h and crystallized from benzene to give hydrazide **7** (58%) as yellow crystals, m.p. 191–193 °C. IR (KBr): (ν/cm^−1^) = 3198 (NH), 1682, 1736 (C=O). ^1^H-NMR (DMSO-*d_6_*) spectra δ_H_ (ppm) = 9.67 (s, 1H, NH), 7.88 (t, *J* = 0.5 Hz, 1H, N=CH), 7.81–7.77 (m, 1H, CH=C), 7.61–7.55 (m, 2H, Ar-H), 7.20–7.14 (m, 1H, Ar-H), 7.12–7.08 (m, 2H, Ar-H), 7.08 (d, *J* = 6.9 Hz, 1H, Ar-H), 7.02 (d, *J* = 8.9 Hz, 1H, Ar-H), 6.01 (s, 2H, dioxole ring), 3.80 (s, 3H, OCH_3_). ^13^C NMR(DMSO-*d_6_*) spectra δ_C_ (ppm) = 169.93, 168.41, 165.85, 161.47, 149.34, 148.33, 145.22, 130.68, 128.79, 128.12, 126.76, 125.32, 119.76, 114.41, 110.52, 109.65, 101.85, 55.35, 42.01. Anal. Calc’d for C_21_H_17_N_3_O_6_S (439): C, 57.40; H, 3.87; N, 9.56. Found: C, 57.34; H, 3.83; N, 9.51.

### 2.2. Computational Study

The Small Molecule Preparation and Protein Selection

The ligand was constructed and minimized through Density-functional theory “DFT” using B3LYP/6-311G** (Supplementary Materials). Molecular docking was achieved into the active sites of PPAR-*γ* (ID: 2PRG) and *α*-amylase (ID: 2QV4) using MOE-2019. The docking procedures are described in the Supplementary Materials.

### 2.3. Biological Study

#### 2.3.1. Animals

Albino male Wistar rats (150 ± 20 g) were obtained. (Supplementary Materials).

#### 2.3.2. Diabetes Induction 

The animals were kept fasting overnight (12 h) before induction of diabetes. Diabetes was induced in rats as reported [27,28,29,30,31] and (Supplementary Materials).

#### 2.3.3. Determination of the BGLs 

Blood samples were obtained from the tail vein of all rats, the blood glucose levels (BGLs) were measured and monitored in all treatment groups at days 0 (basal), 2, 4, 15 and 30 as reported [32].

#### 2.3.4. Biochemical Analysis

The blood samples (0.5 mL) were withdrawn from each rat using the retro-orbital plexus and the other steps for biochemical analysis were adapted. (Supplementary Materials). 

#### 2.3.5. The In Vitro Inhibition of α-Amylase Assay

The *α*-amylase activity of the probe compounds was determined using the method at [33].

#### 2.3.6. Radical Scavenging by DPPH Method

The assay of 2,2-Diphenyl-1-picrylhydrazyl radical (DPPH) was performed according to the method at [34]. 

### 2.4. Statistical Analysis

The analysis of data is described in the Supplementary Materials.

## 3. Results

### 3.1. Chemistry

**Scheme 1:**
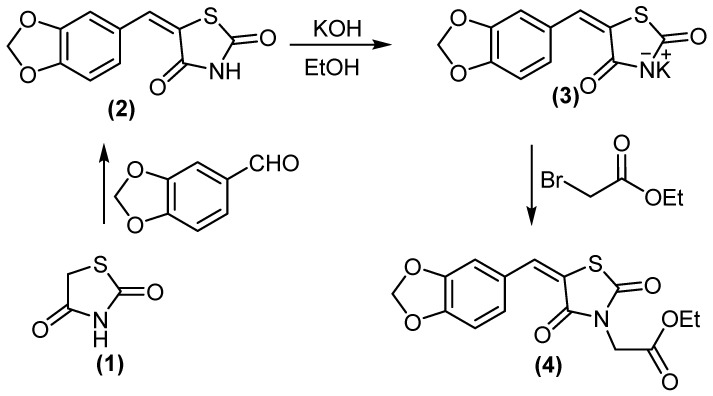
Synthesis of compounds **3** and **4**.

In a continuation of our previous work [27], we reported the synthesis of novel antidiabetic thiazolidinediones. 5-(benzo[*d*][1,3]dioxol-5-ylmethylene)thiazolidine-2,4-dione (**2**) treated with KOH/DMF gave potassium salt derivative (**3**). Molecule **3** was reacted with ethyl bromoacetate to afford ethyl 2-(5-(benzo[*d*][1,3]dioxol-5-ylmethylene)-2,4-dioxothiazolidin-3-yl)acetate (**4**), (Scheme 1). Compound **4** displayed two absorption bands attributed to 2 C=O at 1692 and 1736 cm^−1^, while its ^1^H NMR spectrum (DMSO-*d_6_*) revealed the signal at δ_H_ = 4.18 ppm (2H, CH_2_).

**Scheme 2:**
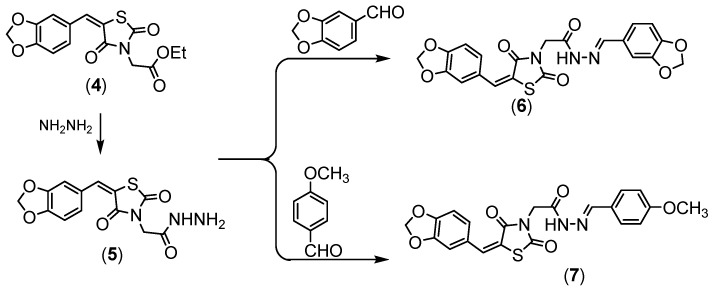
Synthesis of compounds (**5**–**7**).

Compound **4** was hydrazinolyzed to give corresponding hydrazide derivative **5**, (Scheme 2). Compound **5** succeeded in condensation with different aldehyde **5** and **6** derivatives, (Scheme 2). Compound **5** showed the absorption bands attributed to amino group at 3251 and 3147 cm^−1^, the ethoxy resonances disappeared in NMR proton due to the appearance of the amino group at δ = 7.10 ppm.

All the newly obtained thiazolidinediones were separated in good yields, with high purity when applied to HPLC analysis. The chemical structures were fully elucidated using IR, NMR and MS techniques.

### 3.2. Molecular Modeling Studies

#### 3.2.1. Interaction Stability Based on FMO Analysis

**Figure 2:**
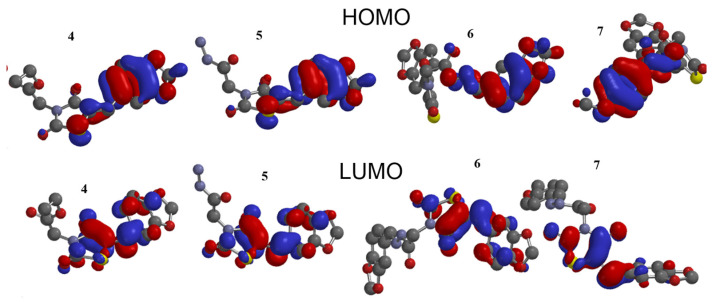
HOMO and LUMO orbitals density for compounds (**4**–**7**).

The stability of interactions (inter- and intra-molecular) between kinase and thiazolidinedione based on FMO were examined used DFT/B3YLP/6311G** as implemented in Gaussian 09 package [35]. The FMOs included HOMO/LUMO (donating electron/accepting electron) can decide the interaction rout between compounds and kinase (Figure 2). The FMOs gap was used to examine the chemical reactivity of the molecule [36]. The growing level of HOMO energy reflects the powerful ability for providing electrons with high susceptibility to oxidation, and vice versa [37,38]. The value for E_HOMO_ was sorted by decreasing order **5** > **7** > **6** > **4** (Table 1). The HOMO sector localized over benzodioxol in all compounds, at the same time the LUMO cloud condensed over benzodioxol ring. 

The HOMO and LUMO have a negative energy indicating the intramolecular electron cloud transfer as thiazolidine-2,4-dione→benzodioxole. The energy gap ΔG is vital for examination of the stability for the biomolecule in the receptor. The low value of the ΔG for the tested compounds postulates a promising interaction between FMO and kinase [37]. 

The chemical reactivity for **4**–**7** was computed in (Table 1), like; η, hardness (quantifies the resistance to change in the electron distribution in a collection of nuclei and electrons); S, softness (reciprocal of hardness) [39]; µ, chemical potential; χ, electronegativity strength [40]; the μ−, electron donating power; μ+, the amount of catching electron; ω−, electron donating capacity; ω+, the amount of electron accepting; ω^±^, net electrophilicity (a relative power between electron accepting and electron donating) [41]; ωI, electrophilicity index in ground state; ωi^VS^, electrophilicity index in valance state [42]. These parameters represented in terms I, ionization potential and A, electron affinity [41]. The amount of electron was measured by reactivity index term “ΔN_max_”, which represented as a stabilization energy, when a system acquired an additional electronic charge from the environment during chemical reaction, the previous terms represented in Equations (S1)–(S11), Supplementary Materials.

The soft nucleophiles and hard electrophiles connected with the raising ΔG, that can measure the stability index (the low energy-gap molecule has improved the polarizability, softness, chemical reactivity and nucleophilicity), and vice versa. Low softness values (~−4.9–5.21 ev.) for the synthesized compounds (**4**–**7**) related to its high reactivity via biological environment (Table 1).

All compounds (**4**–**7**) showed almost similar (ω^+^ and μ^+^) values. The data proved that the ester and hydrazide fragments can improve electron donation to kinase. In addition, these compounds (**4**–**7**) have a very promising electrophilicity value (ω^±^ = 1.27 to 1.3 ev), that figured a good electrophilicity of **4**–**7**, which may enhance the chances of attack over a polar-active site of the receptor.

#### 3.2.2. The MEP Analysis

**Figure 3:**
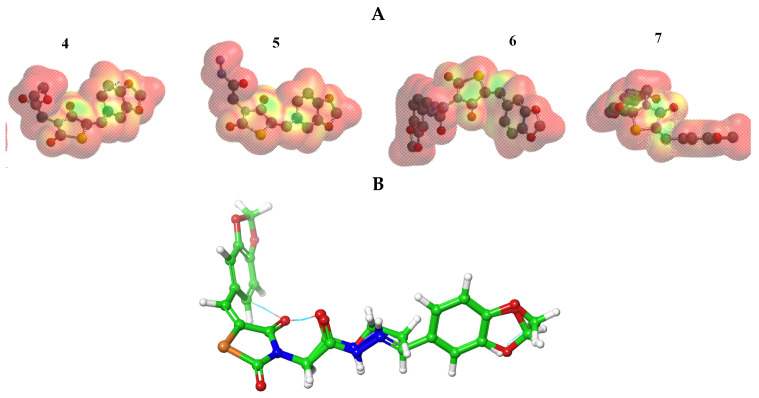
(**A**) MEP plot of synthesized compounds (**4**–**7**); (**B**) Overlapping the chemical structures for all compounds (**4**–**7**).

The MEP was mapped to figure the balance between repulsive interaction (nucleophilic ability) and attractive interaction (electrophilic reactivity) (Figure 3). The orange, yellow and red colors depict the negative power (great electron density area). The colors near to blue shift figured a positive potential, and green color represented intermediate potential ability. MEPs showed that the electron density covered over backbone for compounds (**4**–**7**). The expansion of a red region for these compounds related to electrophilic potency. The variety in shading of MEPs is related to diversity of electrostatic potential values that can be attributed to expanding electrostatic interactions’ ability and identification between substrate and receptor [43]. Furthermore, the conformational analysis and optimization geometry for compounds (**4**–**7**) were performed at DFT and mapped (Figures S1–S4, Supplementary Materials. Thiazolidine-2,4-dione ring is stabilized with benzo[*d*][1,3]dioxole in planarity modes for compounds (**4** and **5**) and is perpendicular in case of compounds (**6** and **7**). From Figure 3B, one can observe the same spatial arrangement of the chemical structures (**4**–**7**).

### 3.3. Docking Studies

The molecular docking was performed via PPAR-*γ* and *α*-amylase to examine a regulation action for compounds (**4**–**7**). The ligand–protein interaction was estimated based on the gold score function in MOE.2019 [44]. From the X-ray of kinases and its regulators [33,45], we can notice that the TZDs to be a kinase-regulator should include the following structural elements: TZD head, lipophilic side chain and aromatic linker. The probe compounds docked successfully into the reported active sites (CYS 285, GLu233, HIS 449, SER299, HIS323, CYS285,ASP300, Arg195), (Asp300, Glu233 and Asp197) for (PPAR-*γ*, PDB: 2PRG [46]) and (*α*-amylase, PDB:2QV4 [47]), respectively. Then, the (MMFF94) force field was applied for optimization of the docked pose. To evaluate the binding affinity “BE” for the tested TZs, we used the highest MOE scoring function with the lowest RMSD (Table 2). For further confirmation of the BE we examined the inhibition constant (Ki) [48], the bioactivity factor and the Ligand-Efficiency (LE) [49]. 

In **PPAR-*γ***: Compounds (**4** and **5**) have the highest BE (−8.25 and −8.43 Kcal/mol.), with promising Ki (0.12 and 0.11 μM), respectively (Table 2). Compound **6** has BE higher than **7**. All compounds showed a normal range of Ki and LE bioactivity parameters as [50]. All compounds (**4**–**7**) interacted with kinase in the same manner for original inhibitor. 

**Figure 4:**
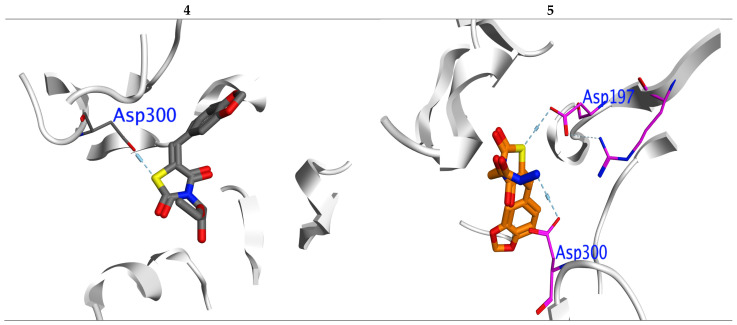
Interaction between ligands **4** and **5** with 2QV4 binding sites in which blue lines represent hydrogen bonding interaction.

In ***α*-amylase:** Compounds (**4** and **5**) also displayed the highest BE (−7.32 and −7.42 Kcal/mol.) with lowest inhibition constant Ki (0.12 and 0.13 μM), respectively (Table 2). All compounds showed a normal range of bioactivity parameters as Ki and LE [50]. Compounds **4** and **5** interacted with Asp300 residue by formation of H-bond, while compound **5** formed one more H-bond with important amino acid ASP197 (Figure 4).

### 3.4. Biological Study

#### 3.4.1. The In Vitro α-Amylase Inhibitory Profile

**Figure 5:**
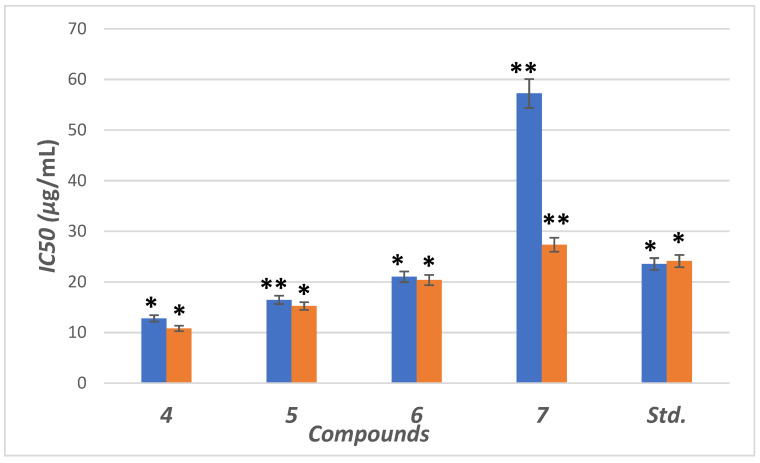
IC_50_ in (μg/mL) for **4**–**7** compared to ascorbic and acarbose, respectively, as standard drugs against DPPH radical (blue column) and *α*-amylase enzyme (orange column), respectively. Data analyzed by one way ANOVA followed by LSD test and expressed as mean ± SEM from five observations; stars represent change as compared to control; ** indicates *p* < 0.01 and * shows *p* < 0.05.

The inhibitory potential was examined for compounds (**4**–**7**) against *α*-amylase enzyme. We used six different concentrations (5, 10, 15, 20, 30 and 40 µg/mL) for this test. The concentrations were plotted against inhibition to determine IC_50_ (concentration required to inhibit 50% of enzyme) using non-linear regression method (Figure 5). Compounds (**4**–**6**) showed higher inhibitory potential (IC_50_ = 11.8 to 21.34 µg/mL) than “STD” standard acarbose (IC_50_ =24.1 µg/mL). Meanwhile **7** with IC_50_ = (57.34 µg/mL) showed lower potency than acarbose with significant (*p* < 0.05) when compared to positive control. The parent compound which bore the ester (**4**), hydrazide (**5**) and benzodioxole (**6**) corners had a more promising activity than acarbose (Figure 5). On the other hand, the inhibition potency decreased by the introduction of the methoxyphenyl fragment to the hydrazone in the molecular skeleton, as in **7**. This efficiency can be attributed to increasing hydrophilicity, which can interact with hydrophobic part of the active site. 

#### 3.4.2. The In Vitro Antioxidant Activity

The antioxidant compounds can protect *β*-cells from reactive oxygen species (ROS) and, therefore, can prevent diabetes induced by ROS [51]. In addition, ROS induces oxidative stress that leads to lipid peroxidation, protein glycation/oxidation and nitration, enzyme inactivation and DNA damage, which leads to the development of various pathological conditions such as diabetes mellitus (DM) and neurodegenerative diseases, which in turn can be neutralized by the presence of endogenous or exogenous antioxidant systems [52,53].

The antioxidant activity was examined for the hybrids (**4**–**7**) using the stable DPPH assay. The antioxidant results from different concentrations (0.16, 0.8, 4, 20, 40, 200 and 1000 µg/mL) were represented in Figure 5 as IC_50_ value (concentration of compound is essential for rising 50% of reducing power for H-donating or radical scavenging). The effect of the scavenging is rising in the trend **4** > **5** > **6** > STD > **7**. Compounds (**4**–**6)** possess antioxidant activity higher than STD (ascorbic acid), while **7** has lower scavenging activity than STD. The compounds with a terminal ester **5** and hydrazide **6** groups showed the highest scavenging potency with IC_50_ = 12.78 and 16.44 μg/mL. After blocking the hydrazide group by aldehyde motifs in compounds **6** and **7**, the activity was decreased. High scavenging activity may be due to the stabilization of the lone pair in the conjugated system by the presence of terminal ester or hydrazide.

#### 3.4.3. The In Vivo Regulation of Blood Glucose Level

Oral anti-hyperglycemic activity was evaluated for compounds **4**–**7** by an alloxan-induced diabetic rat model. Diabetes was successfully induced in the rats under investigation, as verified by their raised blood glucose level (BGL) compared with the normal control rats. The BGL was monitored for each tested compound at three different doses (50,100 and 250 mg/Kg) and for five experimental period durations (0, 2, 4, 15, 30 days).

**Figure 6:**
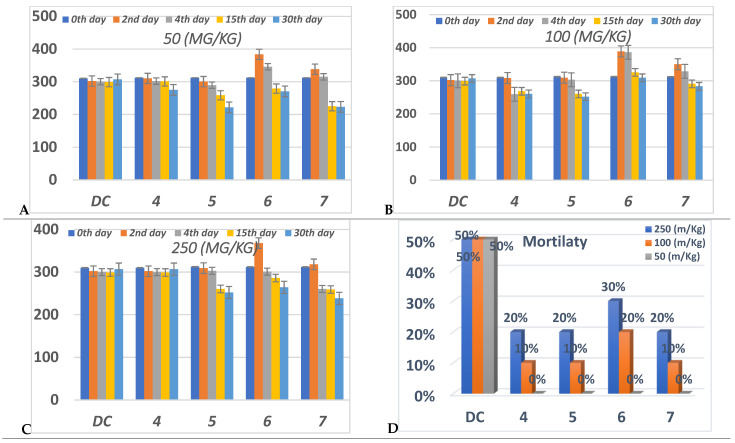
Anti-diabetic effect in three different concentrations, (**A**) 50 (mg/Kg), (**B**) 100 (mg/Kg) (**C**) 250 (mg/Kg), and (**D**) percent of mortality after 30 daysfor each group of compounds (**4**–**7**) in comparison to diabetic control “DC”. Data analyzed by one way ANOVA followed by LSD test and expressed as mean ± SEM from different observations.

All of the compounds **4**–**7** didn’t show obvious reducing of the BGL during the pretreatments compared to DC, while after 30 days, compounds **5**–**7** showed a decrease in the BGL at all concentrations (Figure 6). Compound **7** only reduced BGL significantly during the 4th, 15th and 30th days of the treatments at 250 mg/Kg, while compound **5** organized the BGL more than **6** at these concentrations during the 15th and 30th days. Compound **4** failed to control BGL in all durations of the experiment at 250 mg/Kg compared to the DC.

On the other hand, at 100 mg/Kg concentration, only compounds **4** and **5** achieved a significance decrease in the BGL via DC at the 15th and 30th days; during these days compound **7** displayed an insignificant decrease in the BGL. Compound **6** increased the BGL rather than DC per all of the experiments at 100 mg/Kg.

At 50 mg/Kg concentration; compounds **5**–**7** showed the highest reduction of the BGL at the 15th and 30th days. Compound **7** showed the highest percentage in the BGL reduction at the 15th and 30th days compared to the other members. Conversely, compared to the DC, compounds **4** at the 2nd, 4th and 15th days and **6** at the 2nd and 4th days have suffered from controlling the BGL.

From (Figure 6), all compounds didn’t exhibit any mortality at 50 mg/Kg during the whole experiment.

#### 3.4.4. Lipid Profile

**Figure 7:**
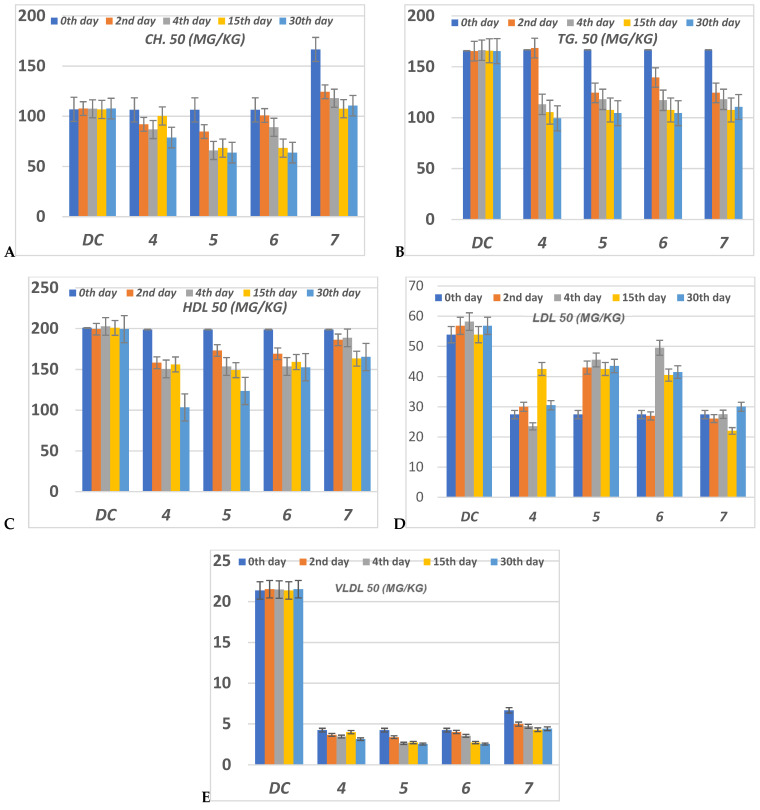
The level of (**A**) CH (Cholesterol); (**B**) TG (Triglyceride); (**C**) HDL (High density lipoprotein); (**D**) LDL (Low density lipoprotein) and (**E**) VLDL (Very low density lipoprotein) in serum of hyperglycemic loaded rats. 50 (mg/Kg) at 0th day (pre-drug values), 2nd and 4th days (post-drug values) for each group of compounds **4**–**7** and compared to diabetic control (DC). Data analyzed by one way ANOVA followed by LSD test and expressed as mean ± SEM from five observations.

The serious side effect related to diabetes is hyperlipidemia [54,55]. The insulin deficiency promotes adipocytes to generate fatty acid, hepatic phospholipids and cholesterol [56,57]. Thus, the levels of cholesterol (CH), triglyceride (TG), high (HDL), low (LDL) and very low (VLDL) density lipoproteins, in serum for alloxan-loaded diabetes have been measured at 50 mg/Kg concentration (Figure 7). All lipid parameters for all tested compounds have (*p* < 0.05) compared to the diabetic control group (Figure 7).

The serum CH level in compounds **4**–**6** has decreased to a lower level than DC value during the 4th, 15th and 30th days of the experiment (Figure 7). Compounds **5** and **6** at the 15th and 30th days showed the best reduction of the CH level. At the 2nd and 4th days, compound **6** showed moderate reduction, while compound **5** showed moderate reduction value at the 2nd day but this value was enhanced during the 4th day. Compound **4** showed moderate regulation of the CH value per all the experimental time. Compound **7** represented the worst CH level from all compounds at all experimental times (Figure 7).

All compounds **4**–**7** during the experimental period succeeded in reducing the TG level compared to DC. The TG level for compounds **4**–**7** was decreased lower than DC animals by the percentage between 0–37.66% along all the experimental periods 0th–30th days. Compound **4** only failed to regulate the TG level lower than the DC level at the 2nd day, while in the other periods for treatment it was the most effective member in lowering CH level to a percentage between 33.77–37.66%. Compounds **5**–**7** were able to decrease the TG level with 16–30% more than DC (Figure 7).

Serum HDL level was improved for all compounds **4**–**7** in contrast to the DC. The HDL value was significantly reduced to a nearly normal level. The best member groups were **4** and **5** in all experimental periods because of their achieving a reduced percentage, about 15% to 50% lower than DC. During the whole period of the experiment, the serum LDL was enhanced to normal level for all group members (Figure 7). Compounds **5** and **6** have monitored the level of serum VLDL to be lower than normal value at 15th and 30th days. All compounds **4**–**7** didn’t regulate VLDL levels to be of normal level at the 0th, 4th and 15th days, but they achieved a promising reduction in LDL compared to DC. Compound **4** had regulated VLDL of infected rats to nearly normal value during the 2nd, 14th and 30th days while at the 0th and 15th days it failed to do so.

## 4. Conclusions

An antihyperglycemic compound, based on the thiazolidinedione scaffold, was synthesized and characterized. The FMOs and global reactivity data indicated that the intramolecular charge transfer takes place between the thiazolidine-2,4-dione and benzodioxole corner in two directions. The MEP for these compounds represented that they have promising electrophilicity, that may enhance the attacking chance over a polar active site of the receptor. The molecular docking into the PPAR-*γ* and *α*-amylase showed the high binding-affinity for the synthesized compounds and may be a suitable regulator for both kinases. The in vitro inhibition potency via the *α*-amylase and scavenging radical were evaluated and represented that the parent compound bearing these corners ester 4, hydrazide 5 and benzodioxole 6 are more efficient than reference drugs. This efficacy decreased in the presence of methoxyphenyl group 7 in the molecular skeleton. The anti-diabetic and anti-hyperlipidemic activities in vitro showed that all of the used concentrations for compounds **4**–**7** didn’t show an obvious reduction in the BGL during pre- and post-treatments compared to DC, while after the 30th days of treatment, they decreased the BGL at all concentrations. The ester 5 hybrid was the most potent regarding the regulation of BGL at different concentrations at the 30th day. All compounds didn’t exhibit any mortality at 50 mg/Kg concentration during the whole experiment. Moreover, all compounds enhanced hyperlipidemic levels nearly to the normal level.

## Figures and Tables

**Table 1:** Energetic global reactivity parameters for compounds (**4**–**7**) at DFT with a B3LYP\6-311G** Basics sets.

	4	5	6	7		4	5	6	7
HOMO	−8.78	−9.27	−9.14	−9.20	ω_i_	−3.087	−3.15	−3.232	−3.215
LUMO	−1.19	−1.11	−1.29	−1.22	µ+	−6.882	−7.123	−7.203	−7.401
ΔG	7.59	8.16	7.85	7.98	µ−	6.24	6.405	6.02	6.05
IP	8.78	9.27	9.14	9.2	ω−	2.8	2.70	3.20	2.902
I	1.19	1.11	1.29	1.22	ω^+^	9.04	9.196	9.536	9.408
A	3.795	4.08	3.925	3.99	ω^±^	1.313	1.272	1.328	1.305
η	0.263	0.245	0.254	0.25	ΔN_max_	2.627	2.544	2.657	2.611
S	−4.985	−5.19	−5.215	−5.21					
χ	3.274	3.3	3.464	3.401					

**Table 2:** Docking energy scores (kcal/mol) derived from the MOE for (**3**–**6**, **9** and **11**).

Cpd.	4	5	6	7
**2PRG**				
ΔΕ	−8.25	−8.43	−6.59	−6.14
RMSD	2.23	2.70	1.98	2.95
E._Int_.	17.12	11.59	−25.84	15.12
E._H.B._	−25.24	−14.25	−20.75	−15.75
L.E	−3.46	−2.08	−3.20	−6.50
Ki(μM)	0.12	0.11	0.13	0.13
**2QV4**				
E__place_	−7.32	−7.46	−6.74	−6.14
RMSD	2.29	1.15	1.95	2.95
E._Int_.	17.94	7.30	−23.29	15.12
E._H.B._	−15.45	−30.40	−24.48	−15.75
L.E	−5.674	−6.487	−3.456	−6.463
Ki(μM)	0.127	0.130	0.117	0.107

ΔE: Free binding energy of ligand-pose; E_place: Free binding energy of ligand-receptor; E.Int.: Binding-Affinity energy of ligand-receptor; H.B.: Energy H-bonding between protein and ligand; RMSD; The root mean square deviation of the docking pose compared to the co-crystal ligand position.

## Data Availability

The data presented in this study are available on request from the corresponding author.

## Supplementary Materials

The following are available online, Figure S1: The geometrical opti-mization for compound 4., Figure S2: The geometrical optimization for compound 5, Figure S3: The geometrical optimization for compound 6., Figure S4: The geometrical optimization for compound 7, Table S1: The conformational analysis for selected theoretical geometrical parame-ters of compound 4; Table S2: The conformational analysis for selected theoretical geometrical parameters of compound 5; Table S3: The conformational analysis for selected theoretical geo-metrical parameters of compound 6; Table S4: The conformational analysis for selected theoretical geometrical parameters of compound 7. Equations (S1)–(S10): Quntum equations.

## Sample Availability

Samples of the compounds are not available from the authors.
