# Supplementary material for: Thiazolidinedione Derivatives: In Silico, In Vitro, In Vivo, Antioxidant and Anti-Diabetic Evaluation
Source: Molecules. 2022 Jan 27;27(3):830. doi: 10.3390/molecules27030830 (PMC8838189; doi:10.3390/molecules27030830)
Supplement: Supplementary file 1 [file molecules-27-00830-s001.zip › molecules-1538934-supplementary.pdf]

Thiazolidinedione Derivatives: In Silico, In Vitro, In Vivo, Antioxidant and Anti-Diabetic Evaluation

Manal Y. Sameeh [1], Manal M. Khowdiary [1,2], Hisham S. Nassar [3,4], Mahmoud M. Abdelall [4], Hamada H. Amer [5,6], Abdelaaty Hamed [4] and Ahmed A. Elhenawy [3,4,*]

1 Faculty of Applied Science, Chemistry Department, Umm El Qura Branch, Lieth, Saudi Arabia; Myassemih@uqu.edu.sa (M.Y.S.); mmkhowdiary@uqu.edu.sa (M.M.K.)
2 Egyptian Petroleum Research Institute, Applied Surfactant Laboratory, Nasr City, Cairo 11727, Egypt.
3 Department of Chemistry, Faculty of Science and Arts in Al-Mukhwah, Al-Baha University, Saudi Arabia; h_nassar5@yahoo.com (H.S.N)
4 Chemistry Department, Faculty of Science, Al-Azhar University, Nasr City-Cairo 11884, Egypt; abdelall_sci@yahoo.com (M.M.A.A.); abdelaatyhamed.1@azhar.edu.eg (A.H.)
5 Department of Animal Medicine and Infectious Diseases, Faculty of Veterinary Medicine, University of Sadat City, Sadat City, Egypt; dr.hamada1435@gmail.com
6 Department of Chemistry, Turabah University College, Taif University, P.O. Box 11099, Taif 21944, Saudi Arabia; h.amer@tu.edu.sa
* Correspondence: ahmed.elheawy@azhar.edu.eg or elhenawy_sci@hotmail.com; Tel.: +966-59-9044526

Spectral instruments:

Melting points were taken on a Griffin melting point apparatus and are uncorrected. Thin layer chromatography (Rf) for analytical purposes were carried out on silica gel and developed. Benzidine, ninhydrin, and hydroxamate tests used for detection reactions. The IR spectra of the compounds were recorded on a Perkin–Elmer spectrophotometer model 1430 as potassium bromide pellets and frequencies are reported in cm-1. The NMR spectra were observed on a Varian Genini-300 MHz spectrometer and chemical shifts (δ) are in ppm. The mass spectra were recorded on a mass spectrometer HP model MS–QPL000EX (Shimadzu) at 70 eV. Elemental analyses (C,H,N) were carried out at the Microanalytical Centre of Cairo University, Giza, Egypt.

Drugs and chemical agents:

Alloxan- monohydrate, Metformin and 2,2-Diphenyl-1-picrylhydrazyl radical (DPPH.) were obtained from Sigma Aldrich Chemical Co. (USA). Reagent kits were obtained from Biodiagnostic, Giza, Egypt. All other chemicals were of the highest available commercial grade.

- *Computational model:*

All the Quantum chemical computations were performed, using the PM3 semi-empirical Hamiltonian molecular orbital calculation MOPAC16 package{Stewart, 1993 #175;Stewart, 1993 #3}, then employing density function theory in Gaussian 09 W program package{Frisch, 2013 #163;Frisch, 2013 #163} with the Becke3-Lee-Yang-parr (B3LYP) level using 6-311G* basis as implemented in MOE 2015 package{Molecular Operating Environment (MOE), #165;Molecular Operating Environment (MOE), #165}. The optimization Geometry for molecular structures were carried out, for improve knowledge of chemical structures. Our compounds were introduced into the binding sites according to the published crystal structures.

- *MOE Stepwise Docking Method:*

The crystal structures complexed with reference inhibitor were obtained. Water and inhibitors molecule were eliminated, and hydrogen atoms were added. The different poses were generated by default triangular-matcher-placement-method. Then refined using AMBER forcefield. Finally, the docking score were obtained based on the free energy as following equation:

$$\Delta G = c + E_{\text{flex}} + \sum_{\text{h-bonds}} c_{\text{HB}} f_{\text{HB}} + \sum_{\text{m-lig}} c_{\text{M}} f_{\text{M}}$$

where c; average gain or loss of rotational/translational entropy; E_{flex}: energy of the lack of flexibility for; fHB: energy for defecting of H-bond geometric .; cHB: energy of ideal H-bond; fM energy of imperfections for metal interaction; cM : energy of an ideal M-bond.

- *inhibition in-vitro of α-Amylase assay:*

The α-amylase activity of the compounds were determined using a method in which 250 µL of each compound, or Metformin .HCl, dissolved in ethanol to obtain different concentrations (5, 10, 15, 20,30 and 40 µg/mL), was mixed with 500 µL of α-amylase (2 U/mL) in phosphate buffer (100 mM, pH 6.8) and incubated at 37 °C for 10 min. Thereafter, 50 µL

of 1% starch, dissolved in the same buffer, was added to the mixture and the samples were incubated again at 37 °C. After 30 min, dinitrosalicylic acid (DNS) colour reagent (1 mL) was added. The (DNS) was prepared by adding 437mg of 3,5- dinitro salicylic acid in 20 mL distilled water to12 g of sodium potassium tartrate in 8 mL of 2 M NaOH, then added distilled water until the solution reach to 40 mL). the samples were boiled for 10 min in water bath. For non-enzymatic reactions, the assays were performed with a blank containing all of the components except the enzyme. Each concentration was analyzed in three independent experiments run in triplicate.
The percent of inhibition Absorbance was then measured at 540 nm. The percent of inhibition of α-amylase was calculated as following by the formula below:
*%inhibition= ((absorbance of blank_absorbance of compound)/absorbance of blank) *100.*

Pharmacological study

- ***Animals:***

Animals. Healthy male albino Wistar rats (200 ± 20 g) were obtained from the Experimental Animal House of the National Research Centre (NRC), Cairo, Egypt, and maintained under controlled conditions (25 °C, 50% air humidity with a 12 h light/dark cycle) with free access to food and water. All animal experiments were carried out under the protocol approved by the Institutional Animal Ethics Committee (Medical Research Ethics Committee (MREC) of the NRC, Cairo, Egypt.

- ***Experimental design.***

Rats were randomly divided into 5 groups of 8 animals each as follows: Group I consisted of healthy rats, to serve as normal control; Group II consisted of diabetic rats representing the diabetic control. Groups III, IV and V were treated with RG marketed tablets and the two selected RG-PLC-M formulations, respectively, in a daily oral dose of 2 mg/kg (Gadadare et al., 2015) for 7 consecutive days, using an oral feeding needle.

- ***Induction of diabetes.***

The animals were kept fasting overnight (12 h) before induction of diabetes. Diabetes was induced in rats by a single subcutaneous injection of alloxan (100 mg/kg) (Fernandes et al., 2007) dissolved in normal saline (Bodade et al., 2013). The animals were allowed to drink 5% glucose solution overnight to overcome the drug induced hypoglycemia (Gandhi and Sasikumar, 2012; Pareek etal., 2009). Blood samples were taken 48 h after injection of alloxan to ensure that diabetes has been induced (Vijayan et al., 2013), and fasting plasma

glucose level of rats was determined using glucose strips. Rats with plasma glucose concentration N 300 mg/dl (Gadadare et al., 2015) were considered diabetic and included in the experiments.

- ***Biochemical analysis.***

The blood samples (0.5 ml) were withdrawn from the retro-orbital plexus of each rat under mild anesthesia using a glass capillary tube after fasting for 6 h. Blood samples were centrifuged at 3000 rpm for 15 min. The serum was kept at −80 °C until analyzed. Specific reagent kits were used to determine levels of serum triglyceride (TG), serum cholesterol (CH), serum low density lipoproteins (LDL), serum high density lipoproteins (HDL) for assessing the lipid profile.

- ***Statistical analysis:***

Data are expressed as mean ± S.E. Data analysis was done using one way analysis of variance (ANOVA) followed by least significant difference (LSD) test for multiple comparisons. Difference was considered significant when *p* is less than 0.05. SPSS (version 11) program was used to carry out these statistical tests.

Quntum equations:

$\eta = 1/2[\partial^2 E/\partial^2 N^2]_{\upsilon(r)} \;=\; 1/2[\partial\, E/\partial\, N^2]_{\upsilon(r)}$	equation	S (1)
$S = \; 1/\eta$	equation	S (2)
$\mu \;=\; [\partial E/\partial N]_{\upsilon(r)}$	equation	S (3)
$\chi = \; -\mu \;= -[\partial E/\partial N]_{\upsilon(r)}$	equation	S (4)
$\mu^{-} = -¼(3I + A)$	equation	S (5)
$\mu^{+} = -¼(I + 3A)$	equation	S (6)
$\omega^{-} = -(3I + A)^2 / 16(I - A)$	equation	S (7)
$\omega^{+} = \quad (3I + A)^2 \;/\; 16(I - A)$	equation	S (8)
$\omega^{\pm} = \omega^{+} + \omega^{-}$	equation	S (9)
$\omega i = \mu^2/2ή$	equation	S (10)
$\omega_i^{Vs} = (I + A)^2 / 4(I - A)$		

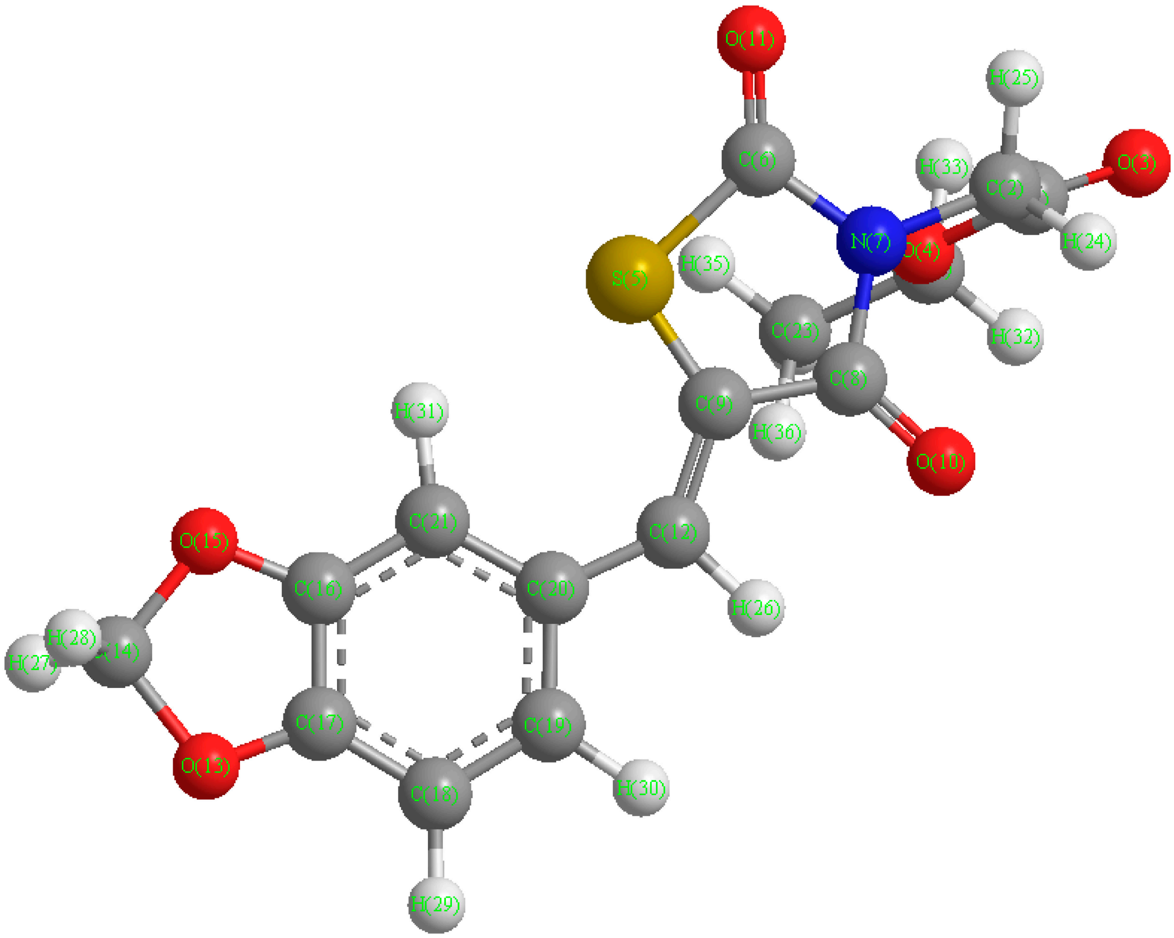

Figure S1. the geometrical optimization for compound 4.

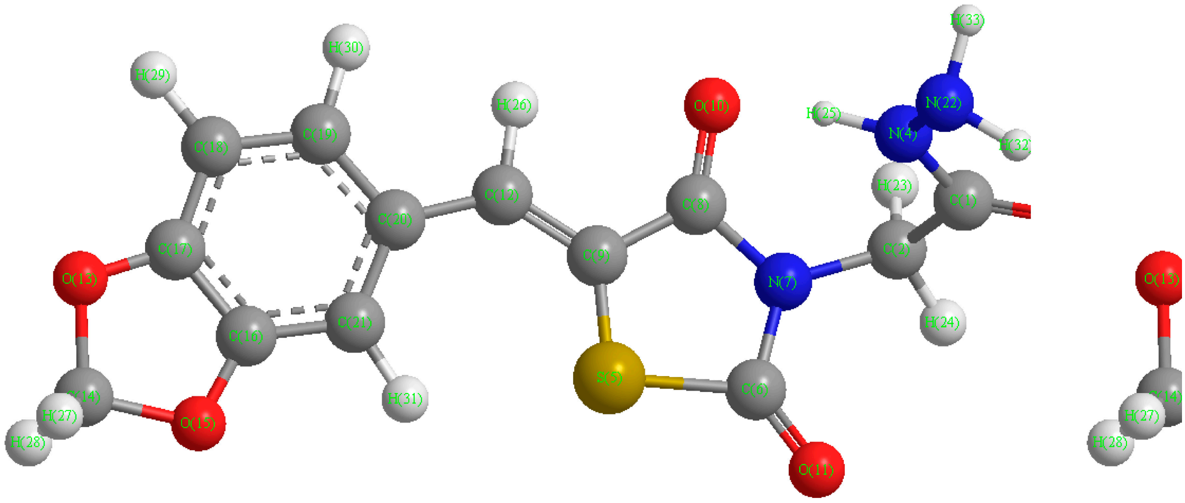

Figure S2. the geometrical optimization for compound 5.

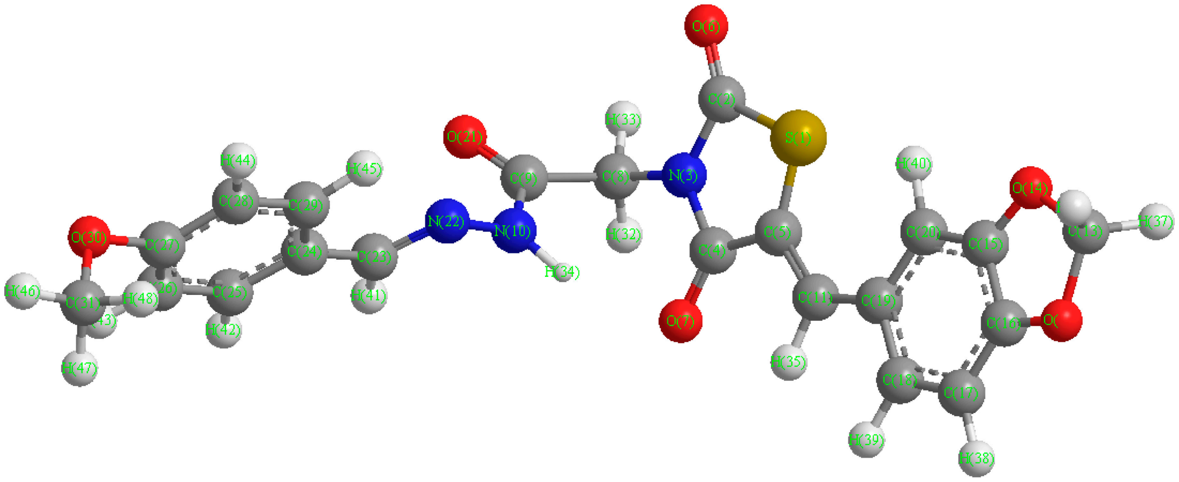

Figure S3. the geometrical optimization for compound 6.

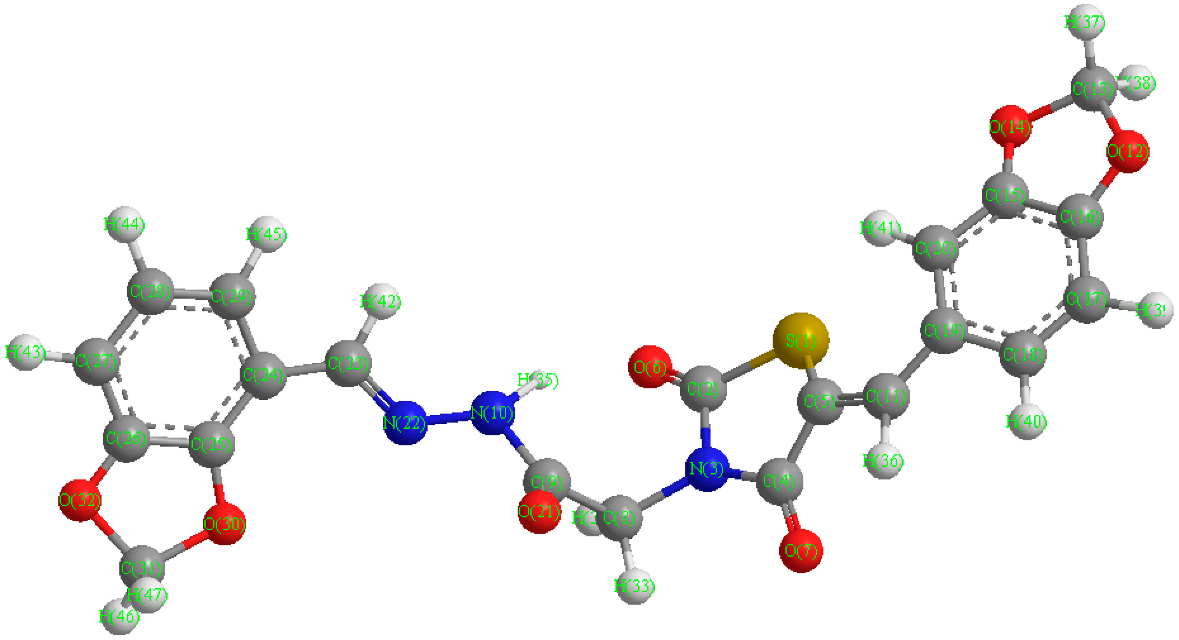

Figure S4. the geometrical optimization for compound 7.

Table S1. The conformational analysis for selected theoretical geometrical parameters of compound *4*.

Atom	Length(Å)	Atom	Angle(°)	Atom	Angle(°)
C(23)-H(36)	1.114	H(36)-C(23)-H(35)	107.86	C(12)-C(9)-C(8)	120.407
C(23)-H(35)	1.114	H(36)-C(23)-H(34)	107.851	S(5)-C(9)-C(8)	113.176
C(23)-H(34)	1.114	H(36)-C(23)-C(22)	111.073	O(10)-C(8)-C(9)	127.504
C(22)-H(33)	1.115	H(35)-C(23)-H(34)	107.848	O(10)-C(8)-N(7)	121.356
C(22)-H(32)	1.115	H(35)-C(23)-C(22)	111.079	C(9)-C(8)-N(7)	111.134
C(21)-H(31)	1.096	H(34)-C(23)-C(22)	110.981	C(2)-N(7)-C(8)	122.32
C(19)-H(30)	1.105	H(33)-C(22)-H(32)	109.851	C(2)-N(7)-C(6)	122.01
C(18)-H(29)	1.102	H(33)-C(22)-O(4)	109.235	C(8)-N(7)-C(6)	115.554
C(14)-H(28)	1.115	H(33)-C(22)-C(23)	110.067	O(11)-C(6)-N(7)	127.534
C(14)-H(27)	1.115	H(32)-C(22)-O(4)	109.305	O(11)-C(6)-S(5)	130.621
C(12)-H(26)	1.106	H(32)-C(22)-C(23)	109.91	N(7)-C(6)-S(5)	101.84
C(2)-H(25)	1.114	O(4)-C(22)-C(23)	108.447	C(9)-S(5)-C(6)	98.224
C(2)-H(24)	1.114	H(31)-C(21)-C(20)	124.262	C(22)-O(4)-C(1)	117.95
C(20)-C(21)	1.351	H(31)-C(21)-C(16)	115.223	H(25)-C(2)-H(24)	105.7
C(16)-C(21)	1.34	C(20)-C(21)-C(16)	120.515	H(25)-C(2)-N(7)	110.644
C(17)-C(16)	1.335	C(21)-C(20)-C(19)	116.349	H(25)-C(2)-C(1)	107.938
O(15)-C(16)	1.229	C(21)-C(20)-C(12)	126.268	H(24)-C(2)-N(7)	110.669
C(14)-O(15)	1.429	C(19)-C(20)-C(12)	117.382	H(24)-C(2)-C(1)	108.631
O(13)-C(14)	1.428	H(30)-C(19)-C(18)	115.191	N(7)-C(2)-C(1)	112.957
C(17)-O(13)	1.228	H(30)-C(19)-C(20)	121.132	O(4)-C(1)-O(3)	125.449
C(18)-C(17)	1.336	C(18)-C(19)-C(20)	123.678	O(4)-C(1)-C(2)	110.701
C(19)-C(18)	1.345	H(29)-C(18)-C(17)	120.586	O(3)-C(1)-C(2)	123.85
C(20)-C(19)	1.357	H(29)-C(18)-C(19)	121.477		
O(4)-C(22)	1.401	C(17)-C(18)-C(19)	117.937		
C(22)-C(23)	1.526	C(16)-C(17)-O(13)	111.717		
C(2)-N(7)	1.466	C(16)-C(17)-C(18)	120.047		
C(9)-C(12)	1.351	O(13)-C(17)-C(18)	128.236		
C(12)-C(20)	1.356	C(21)-C(16)-C(17)	121.474		
C(6)-O(11)	1.205	C(21)-C(16)-O(15)	127.555		
C(8)-O(10)	1.212	C(17)-C(16)-O(15)	110.971		
S(5)-C(9)	1.481	C(16)-O(15)-C(14)	107.577		
C(8)-C(9)	1.379	H(28)-C(14)-H(27)	110.694		
N(7)-C(8)	1.389	H(28)-C(14)-O(15)	110.838		
C(6)-N(7)	1.369	H(28)-C(14)-O(13)	110.842		
S(5)-C(6)	1.786	H(27)-C(14)-O(15)	110.832		
C(1)-O(4)	1.361	H(27)-C(14)-O(13)	110.851		
C(1)-O(3)	1.212	O(15)-C(14)-O(13)	102.534		
C(1)-C(2)	1.53	C(14)-O(13)-C(17)	107.202		
C(9)-O(21)	1.208	H(26)-C(12)-C(9)	112.78		
C(5)-C(11)	1.351	H(26)-C(12)-C(20)	112.901		
C(11)-C(19)	1.356	C(9)-C(12)-C(20)	134.319		
N(3)-C(8)	1.465	C(12)-C(9)-S(5)	126.413		

Table S2. The conformational analysis for selected theoretical geometrical parameters of compound *5*.

Atom	Length(Å)	Atom	Angle(°)	Atom	Angle(°)
**N(22)-H(33)**	1.019	**H(33)-N(22)-H(32)**	104.687	**O(11)-C(6)-S(5)**	130.302
**N(22)-H(32)**	1.021	**H(33)-N(22)-N(4)**	110.682	**N(7)-C(6)-S(5)**	101.945
**C(21)-H(31)**	1.096	**H(32)-N(22)-N(4)**	110.752	**C(9)-S(5)-C(6)**	98.24
**C(19)-H(30)**	1.105	**H(31)-C(21)-C(20)**	124.243	**H(25)-N(4)-N(22)**	119.646
**C(18)-H(29)**	1.102	**H(31)-C(21)-C(16)**	115.239	**H(25)-N(4)-C(1)**	118.685
**C(14)-H(28)**	1.115	**C(20)-C(21)-C(16)**	120.518	**N(22)-N(4)-C(1)**	121.448
**C(14)-H(27)**	1.115	**C(21)-C(20)-C(19)**	116.349	**H(24)-C(2)-H(23)**	105.343
**C(12)-H(26)**	1.106	**C(21)-C(20)-C(12)**	126.267	**H(24)-C(2)-N(7)**	110.639
**N(4)-H(25)**	1.011	**C(19)-C(20)-C(12)**	117.385	**H(24)-C(2)-C(1)**	109.254
**C(2)-H(24)**	1.114	**H(30)-C(19)-C(18)**	115.192	**H(23)-C(2)-N(7)**	110.312
**C(2)-H(23)**	1.114	**H(30)-C(19)-C(20)**	121.132	**H(23)-C(2)-C(1)**	107.989
**C(20)-C(21)**	1.351	**C(18)-C(19)-C(20)**	123.677	**N(7)-C(2)-C(1)**	112.987
**C(16)-C(21)**	1.34	**H(29)-C(18)-C(17)**	120.584	**N(4)-C(1)-O(3)**	122.104
**C(17)-C(16)**	1.335	**H(29)-C(18)-C(19)**	121.477	**N(4)-C(1)-C(2)**	114.962
**O(15)-C(16)**	1.229	**C(17)-C(18)-C(19)**	117.939	**O(3)-C(1)-C(2)**	122.93
**C(14)-O(15)**	1.429	**C(16)-C(17)-O(13)**	111.722		
**O(13)-C(14)**	1.428	**C(16)-C(17)-C(18)**	120.046		
**C(17)-O(13)**	1.228	**O(13)-C(17)-C(18)**	128.231		
**C(18)-C(17)**	1.336	**C(21)-C(16)-C(17)**	121.471		
**C(19)-C(18)**	1.345	**C(21)-C(16)-O(15)**	127.564		
**C(20)-C(19)**	1.357	**C(17)-C(16)-O(15)**	110.965		
**N(4)-N(22)**	1.351	**C(16)-O(15)-C(14)**	107.581		
**C(2)-N(7)**	1.465	**H(28)-C(14)-H(27)**	110.692		
**C(9)-C(12)**	1.351	**H(28)-C(14)-O(15)**	110.842		
**C(12)-C(20)**	1.356	**H(28)-C(14)-O(13)**	110.85		
**C(6)-O(11)**	1.205	**H(27)-C(14)-O(15)**	110.832		
**C(8)-O(10)**	1.212	**H(27)-C(14)-O(13)**	110.844		
**S(5)-C(9)**	1.481	**O(15)-C(14)-O(13)**	102.532		
**C(8)-C(9)**	1.379	**C(14)-O(13)-C(17)**	107.2		
**N(7)-C(8)**	1.389	**H(26)-C(12)-C(9)**	112.787		
**C(6)-N(7)**	1.37	**H(26)-C(12)-C(20)**	112.918		
**S(5)-C(6)**	1.787	**C(9)-C(12)-C(20)**	134.295		
**C(1)-N(4)**	1.367	**C(12)-C(9)-S(5)**	126.514		
**C(1)-O(3)**	1.208	**C(12)-C(9)-C(8)**	120.421		
**C(1)-C(2)**	1.528	**S(5)-C(9)-C(8)**	113.057		
		**O(10)-C(8)-C(9)**	127.478		
		**O(10)-C(8)-N(7)**	121.129		
		**C(9)-C(8)-N(7)**	111.378		
		**C(2)-N(7)-C(8)**	122.483		
		**C(2)-N(7)-C(6)**	122.188		
		**C(8)-N(7)-C(6)**	115.299		
		**O(11)-C(6)-N(7)**	127.753		

Table S3. The conformational analysis for selected theoretical geometrical parameters of compound *6*.

Atom	Length(Å)	Atom	Length(Å)	Atom	Angle(°)
C(31)-H(48)	1.113	C(9)-N(10)	1.37	H(48)-C(31)-H(47)	110.33
C(31)-H(47)	1.113	C(8)-C(9)	1.528	H(48)-C(31)-H(46)	109.136
C(31)-H(46)	1.113	C(4)-O(7)	1.212	H(48)-C(31)-O(30)	109.992
C(29)-H(45)	1.103	C(2)-O(6)	1.205	H(47)-C(31)-H(46)	109.135
C(28)-H(44)	1.104	S(1)-C(5)	1.481	H(47)-C(31)-O(30)	109.991
C(26)-H(43)	1.103	C(4)-C(5)	1.379	H(46)-C(31)-O(30)	108.216
C(25)-H(42)	1.104	N(3)-C(4)	1.389	C(31)-O(30)-C(27)	113.466
C(23)-H(41)	1.105	C(2)-N(3)	1.37	H(45)-C(29)-C(24)	121.204
C(20)-H(40)	1.096	S(1)-C(2)	1.787	H(45)-C(29)-C(28)	117.774
C(18)-H(39)	1.105			C(24)-C(29)-C(28)	121.017
C(17)-H(38)	1.102			H(44)-C(28)-C(29)	119.069
C(13)-H(37)	1.115			H(44)-C(28)-C(27)	118.894
C(13)-H(36)	1.115			C(29)-C(28)-C(27)	122.033
C(11)-H(35)	1.106			C(28)-C(27)-C(26)	116.328
N(10)-H(34)	1.01			C(28)-C(27)-O(30)	121.576
C(8)-H(33)	1.114			C(26)-C(27)-O(30)	121.577
C(8)-H(32)	1.114			H(43)-C(26)-C(27)	118.956
C(24)-C(29)	1.346			H(43)-C(26)-C(25)	119.092
C(28)-C(29)	1.342			C(27)-C(26)-C(25)	121.948
C(27)-C(28)	1.346			H(42)-C(25)-C(26)	118.091
C(26)-C(27)	1.346			H(42)-C(25)-C(24)	120.799
C(25)-C(26)	1.342			C(26)-C(25)-C(24)	121.105
C(24)-C(25)	1.346			C(29)-C(24)-C(25)	117.331
C(19)-C(20)	1.351			C(29)-C(24)-C(23)	122.182
C(15)-C(20)	1.34			C(25)-C(24)-C(23)	120.48
C(16)-C(15)	1.335			H(41)-C(23)-C(24)	119.117
O(14)-C(15)	1.229			H(41)-C(23)-N(22)	114.927
C(13)-O(14)	1.429			C(24)-C(23)-N(22)	125.956
O(12)-C(13)	1.428			C(23)-N(22)-N(10)	121.806
C(16)-O(12)	1.228			H(40)-C(20)-C(19)	124.25
C(17)-C(16)	1.336			H(40)-C(20)-C(15)	115.234
C(18)-C(17)	1.345			C(19)-C(20)-C(15)	120.516
C(19)-C(18)	1.357			C(20)-C(19)-C(18)	116.348
O(30)-C(31)	1.41				
C(27)-O(30)	1.371				
C(23)-C(24)	1.347				
N(22)-C(23)	1.264				
N(10)-N(22)	1.353				
C(9)-O(21)	1.208				
C(5)-C(11)	1.351				
C(11)-C(19)	1.356				
N(3)-C(8)	1.465				

Table S4. The conformational analysis for selected theoretical geometrical parameters of compound 7.

Atom	Length(Å)	Atom	Length(Å)	Atom	Angle(°)	Atom	Angle(°)
C(31)-H(47)	1.101	C(9)-N(10)	1.431	C(26)-O(32)-C(31)	100.685	C(15)-C(16)-O(12)	108.973
C(31)-H(46)	1.082	C(8)-C(9)	1.565	H(47)-C(31)-H(46)	111.617	C(15)-C(16)-C(17)	117.315
C(29)-H(45)	1.128	C(4)-O(7)	1.157	H(47)-C(31)-O(32)	109.079	O(12)-C(16)-C(17)	133.696
C(28)-H(44)	1.116	C(2)-O(6)	1.2	H(47)-C(31)-O(30)	110.959	C(20)-C(15)-C(16)	118.881
C(27)-H(43)	1.13	S(1)-C(5)	1.477	H(46)-C(31)-O(32)	107.228	C(20)-C(15)-O(14)	127.514
C(23)-H(42)	1.086	C(4)-C(5)	1.406	H(46)-C(31)-O(30)	114.13	C(16)-C(15)-O(14)	113.605
C(20)-H(41)	1.135	N(3)-C(4)	1.399	O(32)-C(31)-O(30)	103.327	C(15)-O(14)-C(13)	104.498
C(18)-H(40)	1.12	C(2)-N(3)	1.392	C(31)-O(30)-C(25)	108.508	H(38)-C(13)-H(37)	108.721
C(17)-H(39)	1.232	S(1)-C(2)	1.793	H(45)-C(29)-C(24)	124.32	H(38)-C(13)-O(14)	107.246
C(13)-H(38)	1.127			H(45)-C(29)-C(28)	119.33	H(38)-C(13)-O(12)	111.785
C(13)-H(37)	1.131			C(24)-C(29)-C(28)	116.202	H(37)-C(13)-O(14)	112.865
C(11)-H(36)	1.19			H(44)-C(28)-C(27)	119.8	H(37)-C(13)-O(12)	112.241
N(10)-H(35)	1.329			H(44)-C(28)-C(29)	115.922	O(14)-C(13)-O(12)	103.857
C(8)-H(34)	1.113			C(27)-C(28)-C(29)	123.792	C(13)-O(12)-C(16)	106.787
C(8)-H(33)	1.117			H(43)-C(27)-C(28)	120.699	H(36)-C(11)-C(5)	107.96
C(28)-C(27)	1.362			H(43)-C(27)-C(26)	121.023	H(36)-C(11)-C(19)	124.659
C(26)-C(27)	1.316			C(28)-C(27)-C(26)	118.041	C(5)-C(11)-C(19)	127.378
C(25)-C(26)	1.326			C(27)-C(26)-C(25)	116.759	H(35)-N(10)-N(22)	127.529
O(32)-C(26)	1.195			C(27)-C(26)-O(32)	117.658	H(35)-N(10)-C(9)	103.217
C(31)-O(32)	1.413			C(25)-C(26)-O(32)	123.933	N(22)-N(10)-C(9)	118.475
O(30)-C(31)	1.49			C(26)-C(25)-O(30)	102.807	O(21)-C(9)-N(10)	122.267
C(25)-O(30)	1.239			C(26)-C(25)-C(24)	124.037	O(21)-C(9)-C(8)	119.851
C(24)-C(25)	1.339			O(30)-C(25)-C(24)	133.097	N(10)-C(9)-C(8)	117.87
C(29)-C(24)	1.321			C(25)-C(24)-C(29)	118.9	H(34)-C(8)-H(33)	109.191
C(28)-C(29)	1.343			C(25)-C(24)-C(23)	135.728	H(34)-C(8)-N(3)	112.433
C(19)-C(20)	1.348			C(29)-C(24)-C(23)	104.569	H(34)-C(8)-C(9)	108.011
C(15)-C(20)	1.344			H(42)-C(23)-C(24)	126.082	H(33)-C(8)-N(3)	104.893
C(16)-C(15)	1.354			H(42)-C(23)-N(22)	106.902	H(33)-C(8)-C(9)	108.436
O(14)-C(15)	1.233			C(24)-C(23)-N(22)	126.958	N(3)-C(8)-C(9)	113.72
C(13)-O(14)	1.459			C(23)-N(22)-N(10)	127.047	C(11)-C(5)-S(1)	128.749
O(12)-C(13)	1.435			H(41)-C(20)-C(19)	122.581	C(11)-C(5)-C(4)	120.879
C(16)-O(12)	1.251			H(41)-C(20)-C(15)	112.305	S(1)-C(5)-C(4)	108.043
C(17)-C(16)	1.341			C(19)-C(20)-C(15)	120.839	O(7)-C(4)-C(5)	127.818
C(18)-C(17)	1.291			C(20)-C(19)-C(18)	118.15	O(7)-C(4)-N(3)	121.248
C(19)-C(18)	1.372			C(20)-C(19)-C(11)	125.791	C(5)-C(4)-N(3)	110.874
C(23)-C(24)	1.33			C(18)-C(19)-C(11)	115.417	C(8)-N(3)-C(4)	131.595
N(22)-C(23)	1.303			H(40)-C(18)-C(17)	124.012	C(8)-N(3)-C(2)	111.353
N(10)-N(22)	1.341			H(40)-C(18)-C(19)	116.863	C(4)-N(3)-C(2)	115.442
C(9)-O(21)	1.227			C(17)-C(18)-C(19)	119.124	O(6)-C(2)-N(3)	126.298
C(5)-C(11)	1.388			H(39)-C(17)-C(16)	110.767	O(6)-C(2)-S(1)	134.457
C(11)-C(19)	1.357			H(39)-C(17)-C(18)	124.242	N(3)-C(2)-S(1)	99.139
N(3)-C(8)	1.543			C(16)-C(17)-C(18)	123.774	C(5)-S(1)-C(2)	100.677
